# Thoughts on Fang-Filling

**Published:** 1859-07

**Authors:** Geo. Watt


### DENTAL REGISTER OF THE WEST.

VOL. XIII.] JULY, 1859. [No. 1.

### Original Essays and Communications.

### THOUGHTS ON FANG-FILLING.

BY GEO. WATT.

"WHEN the nerves of teeth are destroyed, is it proper to fill the fang and crown, or the crown only?" This question was proposed for discussion at the meeting of the MISSISSIPPI-VALLEY ASSOCIATION, in 1853. As the reader would suppose, a majority, if not all the members present, advocated filling the fangs. At any rate, fang-filling has been *fashionable* since, and probably was before; and the man who claimed that he could fill the fang to the "extreme end" of the canal, could enlist the attention of his fellows, at any time, by proposing to tell them "how to do it." To insure success, it has been considered very desirable, if not essential, that the entire canal be filled.

We propose, now, to say a word in favor of the "crown-only" side of the question—to tell briefly why we believe that fang-filling is not the best practice, in the class of cases under consideration.

If a tooth be filled over a dead nerve or pulp, of course we expect that difficulty will result from the operation. The putrefactive process will continue, with a tendency to extension; and the gaseous products resulting from the decomposition of the dead pulp, will cause sufficient pressure to induce pain and irritation. Increased vitality in the periosteum is manifested,—inflammation is established, which, in such cases, usually terminates in suppuration, an abscess being the final result. It is evident that the inflammation of the periosteum is that which must be guarded against; and what is the most effectual method of preventing this, is the question before us.

As the decomposing matter in the canal is the usual exciting cause, we need be at no loss as to where we are to begin. The dead pulp should be removed,—the canal should be freed from all dead organic matter, whether it be pulp, membrane, mucus, or particles of food. It may be suggested that this is impracticable, and in some cases it is; but it can be done as well if we don't fill the canal with gold as if we do. But we will take it for granted that, after we have done our best, some dead matter still remains in the canal, and will, in view of this, detail our treatment.

We cleanse out the canal as well as practicable, and then arrest the putrefactive process, and fill the cavity of decay, leaving the canal unfilled. The putrefaction may be arrested by several methods. Any agent which will form, with the dead matter, a permanent and insoluble compound, will answer the purpose, provided it exerts no injurious influence on the tooth. If we had to rely on but one agent, we would prefer creosote. It has all the chemical properties which are required. Tannin answers the same purpose, but accomplishes the end more slowly. Chloroform will answer, but is so volatile that it soon passes away, thus rendering frequent applications necessary. Chloride of zinc and nitrate of silver may be used; but the compounds they form with albuminous matter are less permanent than those formed by creosote or tannin.

Having cleansed out the canal, then, we insert a pledget of cotton, moistened with creosote, and let it remain a day or two, with the cavity of decay merely closed with dry cotton. We then renew the application, and confine the creosote in the canal, by filling the cavity with wax, gutta percha, or something similar. The object of this is to retain the vapor of the creosote in the tooth, that it may permeate all parts of the canal. After a day or two, we remove the applications, wash out the cavity with water, and afterward with chloroform, dry it, and fill as above stated.

As there is nothing left in the canal that can putrefy, no danger need be apprehended from this source. And as to the foramen in the end of the fang, nature closes it up better than can be done with gold.

When the canal is filled throughout its entire length with gold, the fang, (and, of course, the periosteum,) is subject to sudden changes of temperature, on account of the conducting power of the gold; and this we believe to be a fruitful source of dental periostitis. In the method we adopt, this is obviated.

Since adopting this course, our success is much better than when we filled the fangs. We frequently tested both methods with the same care, in the mouths of the same patients; and the result is as stated above. We might report a number of cases, but, as we now adopt this method exclusively, they are so numerous, that the few we could find room for would seem insignificant, in comparison with the whole number.

This article, thus far, was written for the June number of the *Register;* and, since writing it, we have examined a number of cases which, at various dates, were treated as detailed above. We will allude to some of them, without attempting to be formal:

CASE 1.—Miss M. A. Aged 17, bilious-sanguine temperament, health good. The upper centrals and left lateral incisor were badly decayed, the pulps dead, and an abscess at the root of the lateral. These were filled April 14th, 1859, after four days' treatment. At present, June 4th, there is no abscess, and no discomfort.

CASE 2.—Mr. L. O. Aged 30, a quadroon, healthy. Upper first bicuspids decayed, pulps exposed. The pulps were destroyed with arsenious acid, and the teeth filled,

Feb. 19th, 1859, ten days after the first visit. June 1st, both doing well.

CASE 3.—Miss L. F. Aged 15, sanguine temperament, good health. The upper cuspidati decayed, pulps exposed. Destroyed the pulps, and filled, Dec. 11th, 1858, two weeks after the first application of the arsenious acid. Exposure to a storm, two weeks afterward, was followed by periostitis, which terminated in resolution, after active treatment. May 20th, teeth doing well.

CASE 4.—Mrs. F. Aged 29, nervous-sanguine temperament, health delicate. A lower second molar, badly decayed, pulp dead. Treated the case a week, and filled, Jan. 21st, 1859. The tooth was comfortable, May 10th, and had not been otherwise, except a slight soreness a few days after it was filled.

CASE 5.—Miss C. Aged 20, sanguine temperament, health good. Upper first bicuspids decayed, pulps exposed. Destroyed pulps with arsenic, and filled, Nov. 17th, 1858, after ten days' treatment. Teeth remained comfortable till April 20th, 1859, when one was attacked with periostitis, which was subdued without the formation of an abscess.

CASE 6.—Mrs. A. Aged 32, nervous-sanguine temperament, healthy. A first upper bicuspid decayed, pulp dead. Cleaned out the canal, washed it with chloroform, then with creosote, and filled the tooth the same day, Dec. 5th, 1858. This tooth has remained comfortable till this date, June 4th, 1859.

CASE 7.—Mr. B. Aged 21,—in all other respects like Case 6. Filled, Dec. 3d, 1858, and was all right, June 1st, 1859.

CASE 8.—Miss M. Aged 17, sanguine-lymphatic temperament, health not good. Upper central incisors decayed, pulps dead. Treated a week, and filled, Oct. 16th, 1858. Teeth doing well, and had caused no discomfort, June 1st, 1859.

CASE 9.—Mr. J. F. Aged 24, sanguine temperament, healthy. An approximate decay in a superior wisdom tooth, pulp exposed at a small point. Capped and filled, Oct. 2d, 1858. Inflammation and death of the pulp followed. Removed the filling, treated with creosote two weeks, (unable all the time to reach the canals in the fangs,) and filled the central cavity, and the cavity of decay, Dec. —, 1858. June 4th, 1859, the tooth is doing well, and has caused no discomfort.

This report of cases might be greatly extended, but this will suffice. We have not seen a case of abscess, following the treatment here described, in the last year. We are satisfied with our success, and shall continue the practice.

